# Neoadjuvant Savolitinib targeted therapy stage IIIA-N2 primary lung adenocarcinoma harboring *MET* Exon 14 skipping mutation: A case report

**DOI:** 10.3389/fonc.2022.954886

**Published:** 2022-08-16

**Authors:** Meng Fu, Chun-Mei Feng, Da-Qing Xia, Zi-Mei Ji, Huai-Ling Xia, Na-Na Hu, Zai-Jun Leng, Wang Xie, Yuan Fang, Le-Jie Cao, Jun-Qiang Zhang

**Affiliations:** ^1^ Department of Respiratory and Critical Care Medicine, The First Affiliated Hospital of USTC, Division of Life Science and Medicine, University of Science and Technology of China, Hefei, China; ^2^ Anhui Province Key Laboratory of Medical Physics and Technology, Institute of Health and Medical Technology, Hefei Institutes of Physical Science, Chinese Academy of Sciences, Hefei, China; ^3^ University of Science and Technology of China, Hefei, China; ^4^ Anhui Medical University, Hefei, China

**Keywords:** savolitinib, *MET* exon 14 skipping, neoadjuvant therapy, non-small cell lung cancer, targeted therapy

## Abstract

*MET* exon 14 skipping mutation (*MET*ex14m) is rare and occurs in approximately 1-4% of all non-small cell lung cancer (NSCLC) patients and approximately 2.8% of resected stage I-III NSCLC patients. Savolitinib is an oral, potent and highly selective type Ib *MET* inhibitor, which has been shown to be promising activity and acceptable safety profile in patients with advanced NSCLC harboring *MET*ex14m. Most recently, many studies have been probing into the feasibility and efficacy of target therapy for perioperative application in NSCLC. Interestingly, there are very few recorded cases of such treatments. Here, we presented that systemic treatment with the *MET* inhibitor savolitinib before surgery could provide the potential to prolong overall survival (OS) of patients with locally advanced potentially resectable NSCLC. A 49-year-old woman was diagnosed with stage IIIA (T2bN2M0) primary lung adenocarcinoma exhibiting a *MET*ex14m by real-time quantitative polymerase chain reaction (RT-qPCR). Given that the tumor load and the size of lymph nodes experienced a significant downstaging after the neoadjuvant treatment of savolitinib with 600mg once a day for 5 weeks, left lower lobectomy and systemic lymphadenectomy were successfully performed. The pathological response was 50% and the final postoperative pathological staging was pT1cN0M0, IA3 (AJCC, 8^th^ edition). The case provides empirical basis for the neoadjuvant treatment with savolitinib in *MET*ex14m-positive locally advanced primary lung adenocarcinoma, which will offer some innovative insights and clinical evidence for more effective clinical treatment of neoadjuvant targeted therapy for *MET*ex14m-positive NSCLC.

## Introduction

The tyrosine-protein kinase Met (c-Met), also known as hepatocyte growth factor receptor (HGFR), is a heterodimer transmembrane tyrosine kinase receptor encoded by the *MET* proto-oncogene. *MET* is a novel therapeutic target for lung cancer and is closely related to the survival, prognosis and certain drug resistance of lung cancer patients (1). *MET* exon 14 skipping mutation (*MET*ex14m), *MET* kinase domain mutation, *MET* amplification and *MET* fusions are included in *MET* genomic alterations. *MET*ex14m is an independent oncogenic driver occurring in 1%-4% of non-small-cell lung cancer (NSCLC) patients. Patients with *MET*ex14m have a distinct clinicopathology and face-poor prognoses (2).

Several TKIs currently have been approved for advanced NCSLC patients with *MET*ex14m, such as savolitinib, capmatinib and tepotinib. Savolitinib is an oral, potent and highly selective type Ib *MET* inhibitor that has yielded promising activity and acceptable safety profile in patients with pulmonary sarcomatoid carcinoma and other NSCLCs harboring *MET*ex14m (2). Recently, a growing body of research has shown the feasibility of the neoadjuvant targeted therapy for early-stage resectable NSCLC patients with anaplastic lymphoma kinase (*ALK*) fusion gene, epidermal growth factor receptor (*EGFR*) mutations, RET rearrangements and ROS proto-oncogene 1 (*ROS1*) rearrangements (3–6). The fact suggests that the untargeted patients with *MET*ex14m had a shorter disease-free survival (DFS) (7). More importantly, since there have been presently no reported instances of savolitinib as a neoadjuvant treatment for NSCLC patients with *MET*ex14m. Here, we present the first case of stage IIIA-N2 primary lung adenocarcinoma patient harboring *MET*ex14m, who underwent left lower lobectomy and systemic lymphadenectomy resection treatment after receiving neoadjuvant savolitinib targeted therapy. Most encouraging of all, the patient received recovered well postoperatively and had no signs of recurrence during follow-up, which may predict better quality of life and prognosis. The case presented primary clinical evidence that supports the use of neoadjuvant treatment with savolitinib in *MET*ex14m-positive locally advanced primary lung adenocarcinoma.

## Case report

**Figure 1:**
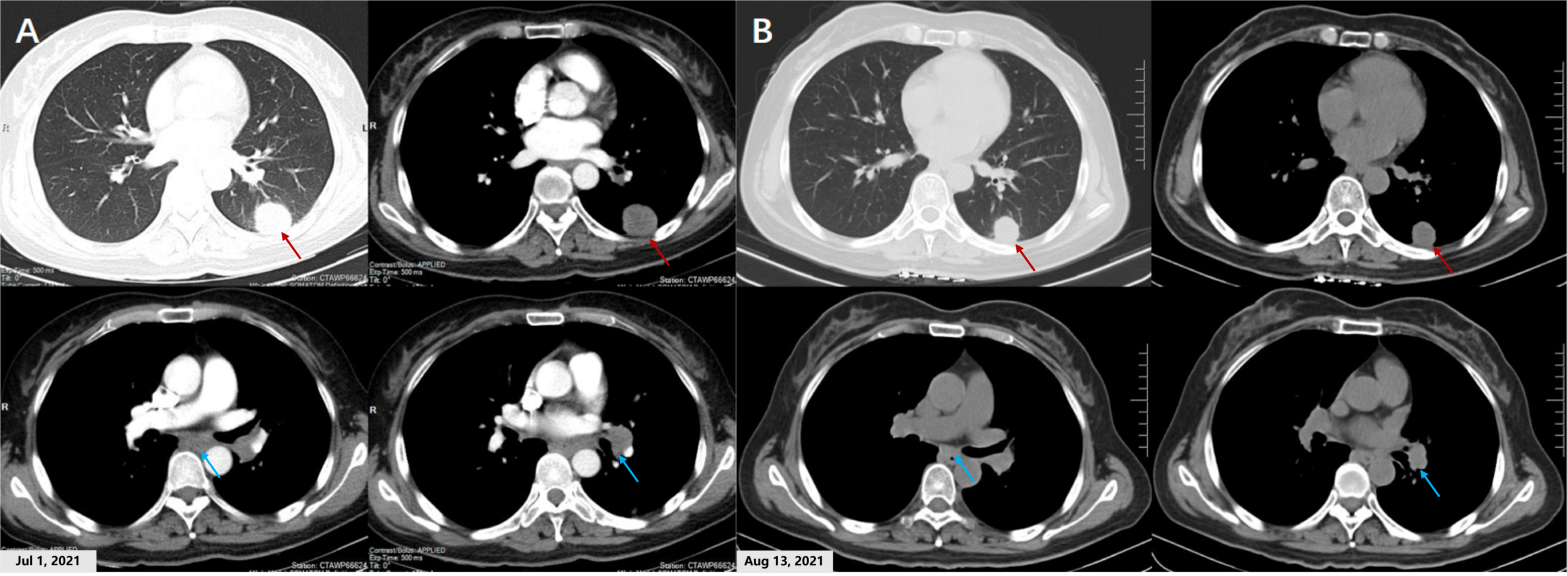
Chest CT scans of the patient before and after savolitinib treatment. **(A)** The chest contrast-enhanced CT scan on July 1, 2021 demonstrates a 3.8 cm × 2.9 cm abnormal lung mass (white arrows) in the lobe of left lung. Enhanced signal was not detected inside the mass. Left hilar and subcarinal lymphadenopathy (red arrows) also were observed. **(B)** After 21 days of savolitinib therapy, the chest CT scan on August 13, 2021 showed the mass (red arrows) had shrunk to 2.6 cm × 2.2 cm, achieving a partial response (PR). The Left hilar and subcarinal lymph node (blue arrows) also shrank significantly, and the short diameter did not exceed 7 mm.

**Figure 2:**
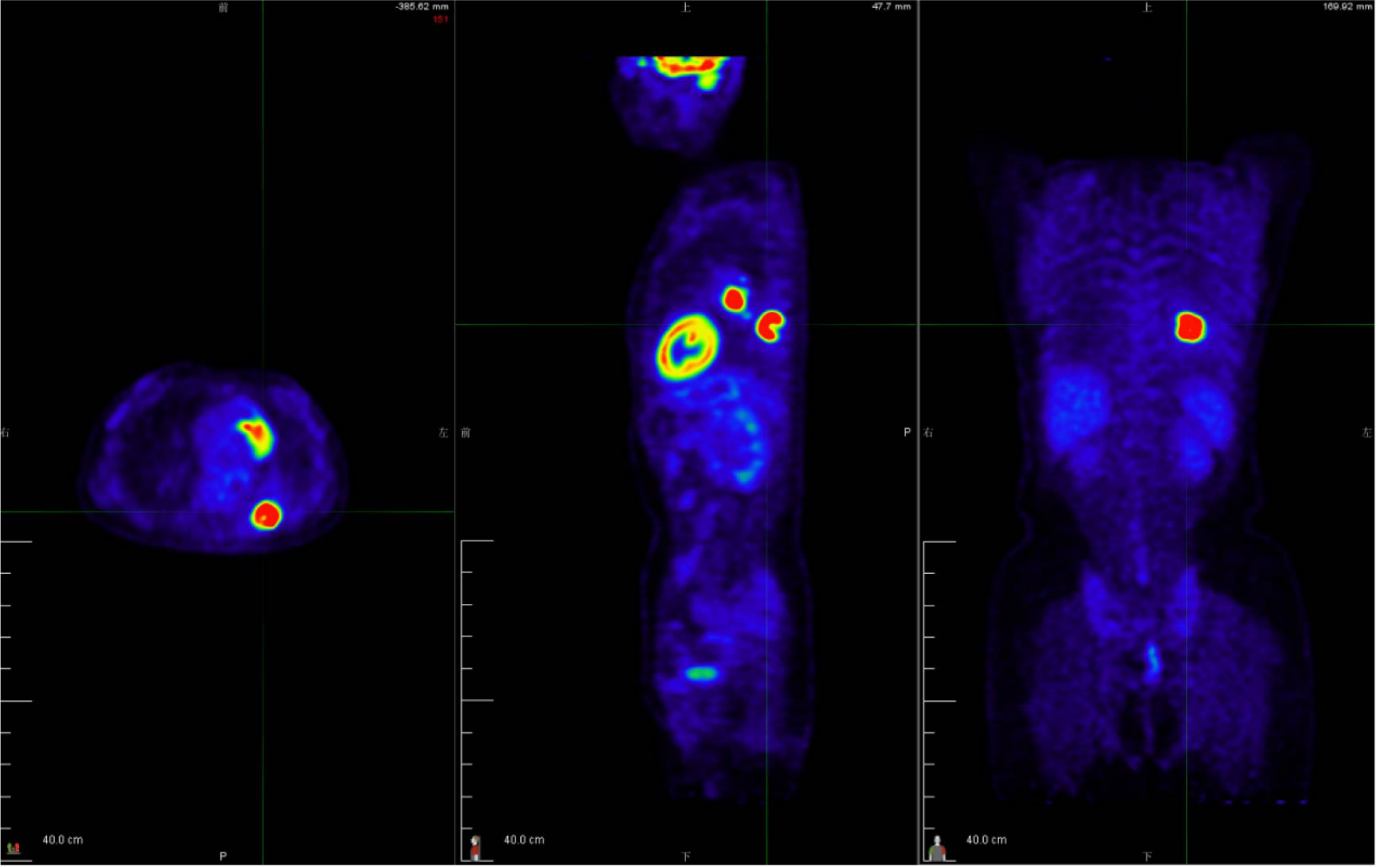
The 18F-FDG PET/CT examination was performed to evaluate the patient’s tumor stage. The 18-FDG PET/CT revealed strong 18-FDG uptake in the left lung mass, as well as the left hilar and subcarinal lymph node, measuring up to 2.5 cm and 2.2 cm in short diameters, respectively, but there was no evidence of distant metastasis. See **Supplementary Material** for a dynamic 3D visualization.

A 49-year-old female patient visited a local municipal tertiary hospital with symptoms including dry cough, chest tightness and voice hoarseness for 1 month. The contrast-enhanced chest CT scan on July 1, 2021 demonstrated a 3.8 cm × 2.9 cm abnormal lung mass in the lobe of left lung. Enhanced signal was not detected inside the mass. Left hilar and subcarinal lymphadenopathy were also observed (**Figure 1A**). Fluorine-18 fluorodeoxyglucose (F18-FDG) positron emission tomography (PET)/CT was performed to evaluate the whole-body situation on July 6, 2021. The 18F-FDG PET/CT showed strong 18-FDG uptake in the left lung mass, as well as the left hilar and subcarinal lymph node, measuring up to 2.5 cm and 2.2 cm in short diameters, respectively, but there was no evidence of distant metastasis (**Figure 2** and **Supplementary Material**). The patient had no history of smoking, no other concomitant diseases, and no family history of cancer.

After initial medical examination and assessment, the patient was admitted to our hospital on July 8, 2021. Stage IIIA (T2bN2M0) lung adenocarcinoma was confirmed on the basis of the 18F-FDG PET/CT results, CT guided lung puncture, and subsequent pathological diagnosis. Genetic tests (real-time quantitative polymerase chain reaction, RT-qPCR) of the lung lesion biopsy revealed the results of *MET*ex14m, whereas other tested driver genes (*EGFR*, *ALK*, *ROS1*, *KRAS*, *BRAF*, *HER2*, *NRAS*, *PIK3CA*, and *RET*) were absent. After active discussions with the multiple disciplinary team (MDT) including respiratory physicians, thoracic surgeons, and radiologists, we all agreed that potentially resectable stage IIIA primary adenocarcinoma after adjuvant therapy would improve prognosis, prolong survival, and improve quality of life. Considering the N2 lymph node metastases involvement, we offered neoadjuvant chemotherapy followed by surgical resection. However, the patient preferred neoadjuvant target therapy rather than neoadjuvant chemotherapy. Given that there was no neoadjuvant indication for *MET* inhibitors, after adequate communication with the patient and her family and receiving written informed consent, we advised the patient to receive the target drug savolitinib as a neoadjuvant therapy based on the results of genomic testing.

**Figure 3:**
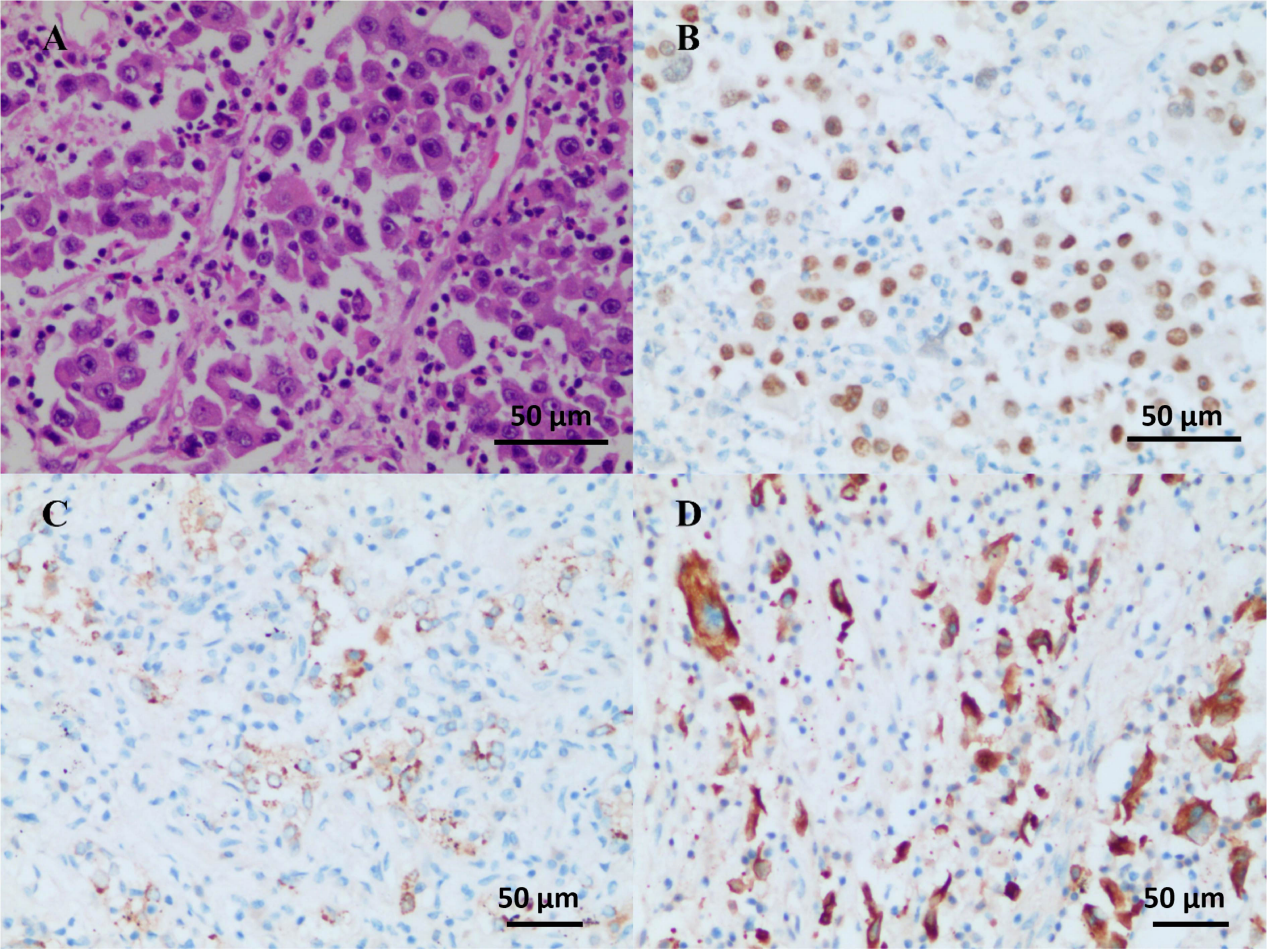
Operative and pathological findings. The pathological diagnosis was poorly differentiated lung adenocarcinoma (solid type) with the size of 2.2 cm × 2.0 cm × 1.2 cm, and the visceral pleura was not involved. No infiltration of cancer cells was detected in the bronchus cutting edges. Pulmonary hilar lymph node (-, 0/5). Immunohistochemistry outcomes were presented as: HE **(A)**, TTF-1 (+) **(B)**, NapsinA (+) **(C)**, CK7 (+) **(D)**, CK5/6 (-), CK (pan) (+), and Ki-67 (+, about 50%).

**Figure 4:**
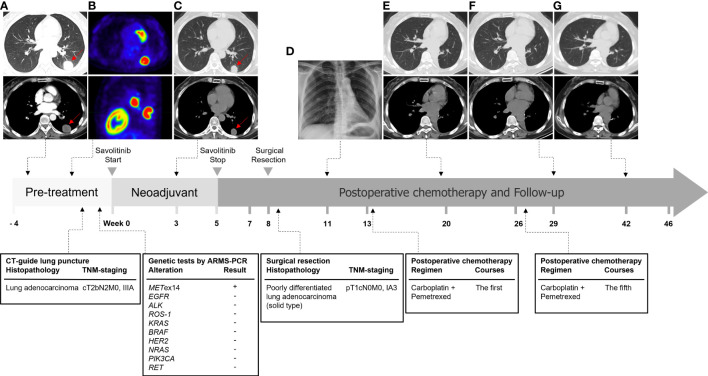
Timeline of diagnosis and treatment of this case. **(A)** The chest contrast-enhanced CT scan at the first visit to the hospital on July 1, 2021. **(B)** The 18-FDG PET/CT examination on July 6, 2021. **(C)** The chest CT scan after 21 days (August 13, 2021) savolitinib neoadjuvant therapy. **(D)** Postoperative chest x-ray on October 13, 2021 revealed good lung expansion of left lung. **(E–G)** The patients underwent re-examination of chest CT on December 20, 2021, March 2, 2022, and June 3, 2022 respectively, which showed postoperative changes and no signs of recurrence.

On July 24, 2021, the patient received savolitinib, 600 mg orally, once daily as neoadjuvant therapy. Around three weeks later (August 13, 2021), the first evaluation of the therapeutic effect presented a partial response (PR) (target lesion shrank from 3.8 cm × 2.9 cm at baseline to 2.6 cm × 2.2 cm), on the basis of the Response Evaluation Criteria in Solid Tumors version 1.1 (RECIST 1.1). Additionally, the left hilar and subcarinal lymph node obviously shrank, and the short diameter did not exceed 7 mm (**Figure 1B**). At the same time, we had also focused on evaluating the patient’s drug tolerance state and adverse drug reactions. The patient experienced grade 2 impaired liver function (ALT 96 IU/L, AST 103 IU/L) during the first 4 weeks after the treatment. Liver enzyme levels in the patient were further increased (ALT 350 IU/L, AST 279 IU/L) after taking diammonium glycyrrhizin enteric-coated capsules for subsequent 5 days. Then, targeted therapy was temporarily discontinued on August 30, 2021 and the patient received glutathione in combination with magnesium isoglycyrrhizinate injection for 2 weeks. On September 14, 2021, the liver function tests returned to normal. There were no other adverse drug reactions such as fatigue, nausea and vomiting, appetite loss, rash, diarrhea, and edema. Considering that radiological downstaging was indicated after 5 weeks of savolitinib treatment, based on the re-discussion of the MDT team, surgical resection of the left lower lung lobe with dissection of the mediastinal lymph nodes was performed on September 24, 2021. The pathological diagnosis was poorly differentiated lung adenocarcinoma (solid type) with a size of 2.2 cm × 2.0 cm × 1.2 cm, and the visceral pleura was not involved. No infiltration of cancer cells was detected in the bronchus cutting edges, achieving R0 resection. The pulmonary hilar lymph node was negative (0/5) (**Figure 3**). The pathological response was 50% and the final postoperative pathological staging pT1cN0M0, IA3 (AJCC, 8^th^ edition). The patient recovered well and the quality of life has been improved accordingly. For patients with completely resected IIIA-N2 stage NSCLC, adjuvant cisplatin-based chemotherapy for 4-6 cycles is recommended to prevent recurrence and improve survival by eradicating minimal residual disease (MRD). Therefore, the patient received following 5 cycles of pemetrexed plus carboplatin adjuvant chemotherapy. Up to now, the patient has been followed up postoperatively for 38 weeks with no sign of recurrence based on the last chest CT examination on June 3, 2022. The timeline for diagnosis and therapeutic interventions for the patient can be seen in **Figure 4**.

## Discussion

Patients with stage I-II NSCLC are generally treated with curative-intent surgery if they are operable. However, some locally advanced (stage III) NSCLC cannot be operated due to factors such as tumor size and/or location. The prognosis of patients with unresectable stage III NSCLC is poor even after concurrent chemoradiotherapy (8). In unresectable stage III NSCLC, despite combination aggressive treatment with radiotherapy and chemotherapy, the 5-year relative survival rate is about 15%-20% (9). Stage III NSCLC is a highly heterogeneous disease and surgical resection with or without neoadjuvant therapy could be carried out in selected patients. Increasing the surgical resection rate is the key link to improving the overall prognosis and survival of patients. Neoadjuvant therapy may improve the overall resection rate and the R0 surgical resection rate of the primary tumor. The potential value of targeted therapy as neoadjuvant therapy for NSCLC patients with specific driver genes has been explored actively. NEOS study (ChiCTR1800016948) evaluated the efficacy and safety of osimertinib as a neoadjuvant treatment in resectable EGFR mutation-positive (EGFRm) lung adenocarcinoma. This study demonstrated the promising efficacy and good tolerability of neoadjuvant osimertinib (10). ESTERN was constructed to provide more insight into the effects on neoadjuvant erlotinib improving operability and survival in EGFRm NSCLC patients with stage IIIA-N2. Among the 19 patients who received erlotinib treatment, 14 patients underwent surgical treatment. The radical resection rate was 68.4% (13/19) with 21.1% (4/19) rate of pathological downstaging. The median progression-free survival (PFS) and overall survival (OS) were 11.2 and 51.6 months respectively in 19 patients with neoadjuvant therapy (11). CTONG 1103 was a randomized controlled phase II trial with erlotinib versus gemcitabine plus cisplatin as neoadjuvant/adjuvant therapy for stage IIIA-N2 EGFRm NSCLC patients. The study showed that neoadjuvant/adjuvant EGFR-TKI has potential and has a promising OS for resected N2 patients with EGFRm NSCLC (12). In a study involved 11 *ALK*-positive patients with pathologically confirmed N2 NSCLC, after received crizotinib at a starting dose of 250 mg twice daily, ten patients received an R0 resection and two patients achieved a pathological complete response to neoadjuvant crizotinib, which provided the evidence for neoadjuvant crizotinib in locally advanced NSCLC (4).

Previous clinical trials have already verified the availability of *EGFR* and *ALK* inhibitors neoadjuvant targeted therapy in the disease control and pathological downstaging for early-stage NSCLC patients harboring the corresponding mutation, and some case reports also observed that target therapy provided effective radiologic and pathologic response in *ALK*, RET and *ROS1*-positive resectable NSCLC patients (3, 6, 13–15). *MET*ex14m is an independent oncogenic driver occurring in 2.8% of resected stage I-III NSCLC patients (16). A previous case report on neoadjuvant treatment with crizotinib in a locally advanced, unresectable *MET*ex14m lung adenocarcinoma achieved pathologic complete response and led to the conversion to resectable disease (17). However, there is no evidence suggests that whether savolitinib can play a role in potentially resectable NSCLC patients. Savolitinib, an oral, highly selective ATP-competitive *MET* inhibitor for treating various cancers including NSCLC, gastric, renal cell carcinoma, esophageal carcinoma, and medulloblastoma, has been approved in China for treating metastatic NSCLC with *MET*ex14m alterations, particularly in patients who fail to tolerate platinum-based chemotherapy or has progress after chemotherapy (18). To date, this is the first case of stage IIIA-N2 *MET*ex14m primary lung adenocarcinoma treated with savolitinib neoadjuvant targeted therapy combined with surgery to reveal noteworthy clinical efficacy. Here, neoadjuvant savolitinib achieved tumor downstage, achieved R0 resection, and even complete the conversion to a potentially curable disease. We found that the patient achieved N0 disease the following 5 weeks of the neoadjuvant savolitinib, whereas 50% of the tumor cells in the postoperative tissues of the patient were still active, which implies the importance of radical resection after the induction of targeted therapy. It is worth noting that drug-induced liver injury (DILI) has been the most common adverse effect of savolitinib during clinical trials and post-market surveillance (19). In this case, the patient experienced grade 3 DILI that led to savolitinib discontinuation after 5 weeks neoadjuvant treatment. After savolitinib withdrawal and liver protection treatment for 2 weeks, the patient’s liver function returned to normal. It is suggested that when using savolitinib, liver function should be monitored carefully.

Additionally, a phase II trial of neoadjuvant and adjuvant capmatinib in patients with stages IB-IIIA, N2, and selected IIIB (T3N2 or T4N2) NSCLC with *MET*ex14m or high *MET* amplification (Geometry-N) is ongoing (NCT04926831) (20). Given the prevalence of the *MET*ex14m in early-stage NSCLC and the preliminary findings from case reports of *MET* inhibitors as neoadjuvant therapies in early-stage NSCLC, clinical trials exploring the role of neoadjuvant *MET* targeted therapies in this population may be warranted.

Overall, the case presented primary clinical evidence that neoadjuvant savolitinib targeted therapy is an effective treatment for *MET*ex14m-positive locally advanced primary lung adenocarcinoma, which can provide a reference for clinical treatment of such patients. Neoadjuvant savolitinib targeted therapy in IIIA-N2 lung adenocarcinoma with *MET*ex14m could achieve pathological downstaging and increase the possibility of radical surgery and R0 resection. Additionally, monitoring liver function is necessary during savolitinib treatment. Along with an acceptable side effect, neoadjuvant targeted therapy probably deserves to be recommended for these patients with lung cancers that harbor a targetable oncogene, which may have much more impressive therapeutic effects than the platinum-based chemotherapy typically used for neoadjuvant therapy. The findings of this case would provide some inspiring insights for prospective clinical studies to further explore the clinical value of neoadjuvant targeted therapy for *MET*ex14m-positive NSCLC.

## Data availability statement

The original contributions presented in the study are included in the article/**Supplementary Material**. Further inquiries can be directed to the corresponding authors.

## Ethics statement

The studies involving human participants were reviewed and approved by the Medical Ethics Committee of the First Affiliated Hospital of University of Science and Technology of China (Anhui Provincial Hospital) (Ethical Approval Number: 2022-RE-056). The patients/participants provided their written informed consent to participate in this study. Written informed consent was obtained from the individual(s) for the publication of any potentially identifiable images or data included in this article.

## Author contributions

L-JC, J-QZ, and MF contributed to the conception and presentation of the case report. L-JC, J-QZ, and D-QX provided clinical expertise and interpretations. L-JC and J-QZ organized the MDT meeting. YF and WX recorded the detail of the MDT meeting. L-JC, J-QZ, H-LX, N-NH, Z-JL, MF, WX, and Z-MJ overall management and treat the patient. MF was responsible for the manuscript writing, literature review, and pictures production. C-MF contributed to manuscript revision, editing and proofreading. All authors contributed to the article and approved the submitted version.

## Funding

This work was supported by the Anhui Provincial Key Clinical Specialty Discipline Construction Program (2021szdzk05). The funding body had no role in the design of the study, and collection, analysis, and interpretation of data and writing the manuscript.

## Acknowledgments

We are thankful to Jing-Jing Kang from the Department of Endocrinology, Fuyang People’s Hospital, who provided of the baseline contrast-enhanced chest CT and PET-CT images of the case. The authors want to thank the colleagues in pathologic and radiologic department for providing the pictures in this case. Finally, we thank the patient for providing consent for this case report.

## Conflict of interest

The authors declare that the research was conducted in the absence of any commercial or financial relationships that could be construed as a potential conflict of interest.

## Publisher’s note

All claims expressed in this article are solely those of the authors and do not necessarily represent those of their affiliated organizations, or those of the publisher, the editors and the reviewers. Any product that may be evaluated in this article, or claim that may be made by its manufacturer, is not guaranteed or endorsed by the publisher.
